# A Novel, Rapid Method to Quantify Intraplatelet Calcium Dynamics by Ratiometric Flow Cytometry

**DOI:** 10.1371/journal.pone.0122527

**Published:** 2015-04-07

**Authors:** Alice Assinger, Ivo Volf, Diethart Schmid

**Affiliations:** Institute of Physiology, Centre for Physiology and Pharmacology, Medical University of Vienna, Austria; Indiana University School of Medicine, UNITED STATES

## Abstract

Cytosolic free calcium ions represent important second-messengers in platelets. Therefore, quantitative measurement of intraplatelet calcium provides a popular and very sensitive tool to evaluate platelet activation and reactivity. Current protocols for determination of intracellular calcium concentrations in platelets have a number of limitations. Cuvette-based methods do not allow measurement of calcium flux in complex systems, such as whole blood, and therefore require isolation steps that potentially interfere with platelet activation. Flow cytometry has the potential to overcome this limitation, but to date the application of calibrated, quantitative readout of calcium kinetics has only been described for Indo-1. As excitation of Indo-1 requires a laser in the ultraviolet range, such measurements cannot be performed with a standard flow cytometer. Here, we describe a novel, rapid calibration method for ratiometric calcium measurement in platelets using both Ar^+^-laser excited fluorescence dyes Fluo-4 and Fura Red. We provide appropriate equations that allow rapid quantification of intraplatelet calcium fluxes by measurement of only two standardisation buffers. We demonstrate that this method allows quantitative calcium measurement in platelet rich plasma as well as in whole blood. Further, we show that this method prevents artefacts due to platelet aggregate formation and is therefore an ideal tool to determine basal and agonist induced calcium kinetics.

## Introduction

Cytosolic free calcium ions [Ca^2+^]_i_ function as important second-messengers in platelets [1]. Diverse autocrine and paracrine stimuli trigger [Ca^2+^]_I_ elevation either by entry of extracellular Ca^2+^ through open channels in the plasma membrane or due to the release of Ca^2+^ from intracellular stores of the endoplasmatic reticulum. The resulting increase in cytosolic [Ca^2+^]_i_ is essential for platelet degranulation via fusion of vesicles with the plasma membrane as well as rearrangement of actin filaments and therefore crucial for platelet shape change and aggregation.

Due to the central role of Ca^2+^ in platelet signalling, measurement of [Ca^2+^]_i_ provides a popular and very sensitive tool to evaluate platelet activation and reactivity. Several fluorescent dyes have been developed to record [Ca^2+^]_i_ dynamics in isolated platelets using cuvette-based methods: In contrast to the fluorescent Ca^2+^ indicator Quin-2 [2] that interferes with platelet function [3], Fura-2 displays a higher quantum yield and allows ratiometric measurements. The crucial advantage of ratiometric measurements is that the obtained results are largely independent of potentially interfering processes such as loading efficiency, efflux or bleaching of the dye or declining instrumental efficiency [4]. Fura-2 can be excited at two different wavelengths (340 and 380 nm) and binding of Ca^2+^ leads to an increase in fluorescence when excited at 340 nm and to a decrease when excited at 380 nm [5]. In contrast, the popular dye Indo-1 allows ratiometric measurements at two emission wavelengths (excitation at 335 nm, emission at 400 and 475 nm).

Flow cytometry has become increasingly important for the measurement of [Ca^2+^]_i_ in platelets and steadily displaces fluorimetric cuvette measurements [6] as it offers several advantages: (i) intraplatelet Ca^2+^can be separated from [Ca^2+^]_i_ of other cells like leukocytes, thereby also allowing experiments in whole blood, (ii) resting platelets can be distinguished from platelet aggregates,and (iii) high numbers of single cellular events are recorded, providing a high statistical power.

Currently quantitative, ratiometric [Ca^2+^]_i_ measurements are performed with Indo-1 labelled platelets employing UV-lasers (e.g. Helium–Cadmium laser, 325 nm), which is not a standard laser on most flow cytometers [7] and does not allow experiments with compounds that exhibit fluorescence when excited in the ultraviolet (UV) range (e.g. oxidized lipids/lipoproteins and/or polyaromatic compounds), which represents a severe drawback of Indo-1.

Co-labelling of platelets with Ca^2+^ indicator Fluo-3 or the brighter and more photostable derivative Fluo-4 [8] with Fura Red [7, 9] provides another option for ratiometric [Ca^2+^]_i_ measurement. Both indicators can be excited simultaneously by a standard blue argon laser and can be recorded in detector channels specific for green (Fluo-3/Fluo-4 emission 488nm) and red (Fura Red emission 637nm) light. Of note, Fluo-3/Fura Red has been shown to be more sensitive compared to Indo-1 [10].

However, to date no appropriate quantitative [Ca^2+^]_i_ measurement of Fluo-4 and Fura Red labelled cells has been described due to the lack of appropriate calibration, as both dyes have dissimilar binding constants (kd values) for Ca^2+^. Applicable calibrations have so far only been published for single ratiometric dyes like Indo-1 and Fura-2 [5].

In this work, we provide a rapid protocol to quantify [Ca^2+^]_i_ in platelets. We describe the mathematical derivation to calibrate [Ca^2+^]_I_ using Fluo-4 and Fura Red in a “two kd model” and confirm that this novel method is suitable for [Ca^2+^]_i_ measurements in platelets. Furthermore, we show that Fluo-4/Fura Red dyes display an extended dynamic range and that the obtained results are unaffected by the formation of platelet aggregates.

## Material and Methods

### Reagents

Adenosine diphosphate (ADP), nigericin, and prostaglandin E_1_ (PGE_1_) were purchased from Sigma Aldrich (Vienna, Austria). Ionomycin was obtained from Fisher Bioreagents (Pittsburgh, PA,USA), carbonyl cyanide m-chlorophenylhydrazone (CCCP) was from Acros Organics (Geel, Belgium). Calcium Calibration Buffer 10 mM CaEGTA and Calcium Calibration Buffer zero (10 mM K_2_EGTA), Fura Red-AM and Fluo-4-AM were obtained from Invitrogen (Lofer, Austria). All concentrations are given as final concentrations.

### Volunteers

All volunteers were free of anti-platelet medication for at least 2 weeks prior to venipuncture. The study was approved by the Human Ethics Committee of the Medical University of Vienna and is in accordance with the Declaration of Helsinki. Informed written consent was obtained from all blood donors.

### Blood collection and preparation of platelet rich plasma and Ca^2+^-free platelets

Blood was drawn with a 20-G needle and anticoagulated with 1/10 volume of 3.2% (w/v) trisodium citrate. To obtain platelet-rich plasma (PRP), blood was centrifuged at 125 g for 20 minutes. To avoid contaminations with other cell types only the upper two thirds of the PRP fraction were used. Buffers and blood preparations were always kept at 37°C and were gassed with 5% CO_2_ to secure stable pH conditions.

Ca^2+^-free platelets were obtained by repeated washing (5 times) of platelets with HEPES-Tyrode buffer (140 mM NaCl, 3 mM KCl, 1 mM MgCl_2_, 16.6 mM NaHCO_3_, 10 mM HEPES, 5.5 mM Glucose, 0.5% human serum albumin, pH 7.4 and gassed with 5% CO_2_) in the presence of PGE_1_ (1 μM) as previously described [11].

### Cell loading with calcium indicators

Whole blood, PRP or Ca^2+^-free platelets in HEPES-Tyrode buffer were incubated with Fluo-4 (3.34 μg/ml; 3.2 μM) and Fura Red (6.67 μg/ml; 6.12 μM) for 20 minutes at 37°C at dark and gassed with 5% CO_2_.

### Ionophores and calibration buffers

Fluorescence labelled isolated platelets, PRP or whole blood were treated with ionomycin (3 μg/ml), nigericin (2 μg/ml) and CCCP (10 μM) and added to calcium free calibration buffer (Buffer A) or a calibration buffer containing 10 mM CaEGTA (Buffer B) to determine fluorescence intensity at maximal and minimal calcium levels. To determine the kd of Fluo-4 and Fura Red, Buffer A and Buffer B were further mixed at different ratios to obtain calcium standard samples ranging from 0 to ~2500 nM free Ca^2+^.To determine the impact of other divalent ions, Mg^2+^ (0.5–1.2 mM), Mn^2+^ (5–20 nM) and Zn^2+^ (5–20 μM) were added to the calibration buffers (see S1 File). Platelets loaded with Ca^2+^ indicators were then added to the different calibration buffers in the presence of ionomycin, nigericin and CCCP and incubated for 30 minutes (37°C, at dark, constantly shaken).To dissect the platelet population in whole blood, platelets were labelled with anti-CD61- Alexa 647 (5 μg/ml, 20 minutes at dark; BioLegend, San Diego, CA, USA). Thereafter, platelets were analysed by flow cytometry.

### Determination of cytosolic free calcium by flow cytometry

Agonist-induced changes in free cytosolic Ca^2+^ in platelets were determined by time-dependent flow cytometry. Therefore fluorescence labelled whole blood or PRP was further diluted (1:50 in HEPES-Tyrode buffer, pH: 7.4) and transferred to a FACS tube, put into the flow cytometer and kept at 37°C by using a self-made tube jacket.

After acquisition of basal Ca^2+^ levels for 50 seconds, the tube was removed to add ADP at different concentrations (1–100 μM) and replaced immediately, so that data acquisition was continued as soon as possible. Flow cytometric analysis was performed with a FACSCalibur flow cytometer (Becton Dickinson, Vienna, Austria). Fluo-4 signals were acquired in FL1 and Fura Red in FL3 using BD Cell Quest Pro Software to obtain FCS2.0 raw data files.

### Data processing

FCS2.0 files exported by Cell Quest Pro Software were directly imported into FlowJo (Tree Star, Ashland, OR, USA) for analysis. To obtain averaged time courses of the fluorescence ratio, an additional parameter FL1/FL3 was defined on the “derived parameter definition” pad with a minimum value of 0.01 before performing kinetic analysis with median statistics. The averaged time series data was subsequently exported to SigmaPlot 12.2 (Systat Software, Chicago, IL). For transformation of ratio data to Ca^2+^ concentrations with the newly developed Equation 10A and 10B (see below), a “user defined transform” was defined and applied to the exported time series data.

### Statistics and data processing

In general results are expressed as means +/-S.E.M. Calibration curves were fitted to the Equations 5, 7A, 7B and 9 using least square minimisation with SigmaPlot 12.2

### Classical “one kd” calibration model

FL1 … (cellular green emission by Fluo-4, autofluorescence corrected)

FL3 …. (cellular red emission by Fura Red, autofluorescence corrected)

r(c)… fluorescence ratio as function of the cytosolic Ca^2+^ concentration,

[Ca^2+^]_i_ = c(r) (cellular cytosolic Ca^2+^ concentration as a function of fluorescence ratio)

Equation 1 shows the classical one kd calibration model based on rationmetric flow cytometric data:

$$r(c)=\frac{FL1(c)}{FL3(c)}$$ (Equation 1)

In cases where c = 0 or c = very high (dyes saturated), ratio is R_min_ or R_max_ as shown in Equation 2 and 3:

$$R_{\min}=\frac{FL1_{\min}}{FL3_{\max}},R_{\max}=\frac{FL1_{\max}}{FL3_{\min}}$$ (Equations 2, 3)

If Fluo-4 and Fura Red were considered as one single dye with two different emission wavelengths in FL1 and FL3 (like Indo-1), Equation 4 would be the ratio as a function of Ca^2+^ concentration [r(c)], and Equation 5 would be [Ca^2+^] as a function of ratio [c(r)], respectively:

$$r(c)=R_{\min}\cdot \frac{R_{\max}-R_{\min}}{c+k_{d}}\cdot \frac{FL3_{\min}}{FL3_{\max}}$$ (Equation 4)

$$c(r)=kd\cdot \frac{r-R_{\min}}{R_{\min}-r}\cdot \frac{FL3_{\max}}{FL3_{\min}}$$ (Equation 5)

This equation has been published previously for Fura-2 and has widely been applied also for Indo-1 measurements [5].

### Newly developed exact “double kd” model

Equation 6A and 6B shows the amounts of dye-1 and dye-2-bound Ca^2+^ (B_1_ and B_2_) as a function of free [Ca^2+^] = c

$$B_{1}=\frac{c\cdot B_{\max\;1}}{c+k_{d1}},B_{2}=\frac{c*B_{\max\;2}}{c+k_{d2}}$$ (Equations 6A and B)

kd_1_, kd_2,_ B_max1,_ B_max2_…mass action binding constants and Bmax values for Ca^2+^ of dye-1 and dye-2, respectively.

This is transformed to fluorescences FL1(c) and FL3(c) given the minimum and maximum fluorescences of both dyes: FL1_min_, FL1_max_, FL3_min_ and FL3_max_ in Equation 7A and 7B:

$$FL1(c)=FL1_{\min}+\frac{c\cdot (FL1_{\max}-FL1_{\min})}{c+k_{d1}},FL3(c)=FL3_{\max}-\frac{c\cdot (FL3_{\max}-FL3_{\min})}{c+k_{d2}}$$ (Equations 7A and B)

The ratio, defined in Equation 1, is expressed in Equation 8

$$r(c)=\frac{FL1_{\min}+\frac{c\cdot (FL1_{\max}-FL1_{\min})}{c+k_{d1}}}{FL3_{\max}-\frac{c\cdot (FL3_{\max}-FL3_{\min})}{c+k_{d2}}}$$ (Equation 8)

Equation 8 can be simplified to Equation 9:

$$r(c)=\frac{c+k_{d2}}{c+k_{d1}}\cdot \frac{c\cdot R_{\max}\cdot FL3_{\min}+k_{d1}\cdot R_{\min}\cdot FL3_{\max}}{c\cdot FL3_{\min}+k_{d2}\cdot FL3_{\max}}$$ (Equation 9)

Rearranging Equation 9 and introduction of h(r), a helper variable as a function of the fluorescence ratio r(c), leads to Equation 10A and 10B, to allow easier expression of c as a function of the ratio in this “double kd” model.

$$h(r)=FL3_{\max}\cdot [k_{d1}\cdot R_{\min}-k_{d2}\cdot r(c)]+FL3_{\min}^{}\cdot [k_{d2}\cdot R_{\max}-k_{d1}\cdot r(c)]$$ (Equation 10A)

$$c(r)=\frac{h(r)-\sqrt{h^{2}(r)+4\cdot FL3_{\min}\cdot FL3_{\max}\cdot k_{d1}\cdot k_{d2}\cdot [R_{\max}-r(c)]\cdot [r(c)-R_{\min}]}}{2\cdot FL3_{\min}\cdot [r(c)-R_{\max}]}$$ (Equation 10B)

## Results

### Development of a “double kd” calibration model

The newly derived mathematical equations for a true “double kd” calibration function are given in the Methods section. The dependence of [Ca^2+^]_i_ on the fluorescence ratio r(c) turned out to be the solution of an implicit quadratic equation. This equation had the additional constant parameters R_min_, R_max_, FL3_min_ and FL3_max_ that had to be obtained from FL1 and FL3 fluorescent data of Ca^2+^-free (R_min_ and FL3_max_) and maximum Ca^2+^ standard samples (R_max_ and FL3_min_), respectively. Kd_1_, kd_2_ were the apparent cellular kd values of the two dyes that were determined as follows:

### Apparent cellular kd values of Fluo-4 and Fura Red

**Fig 1:**
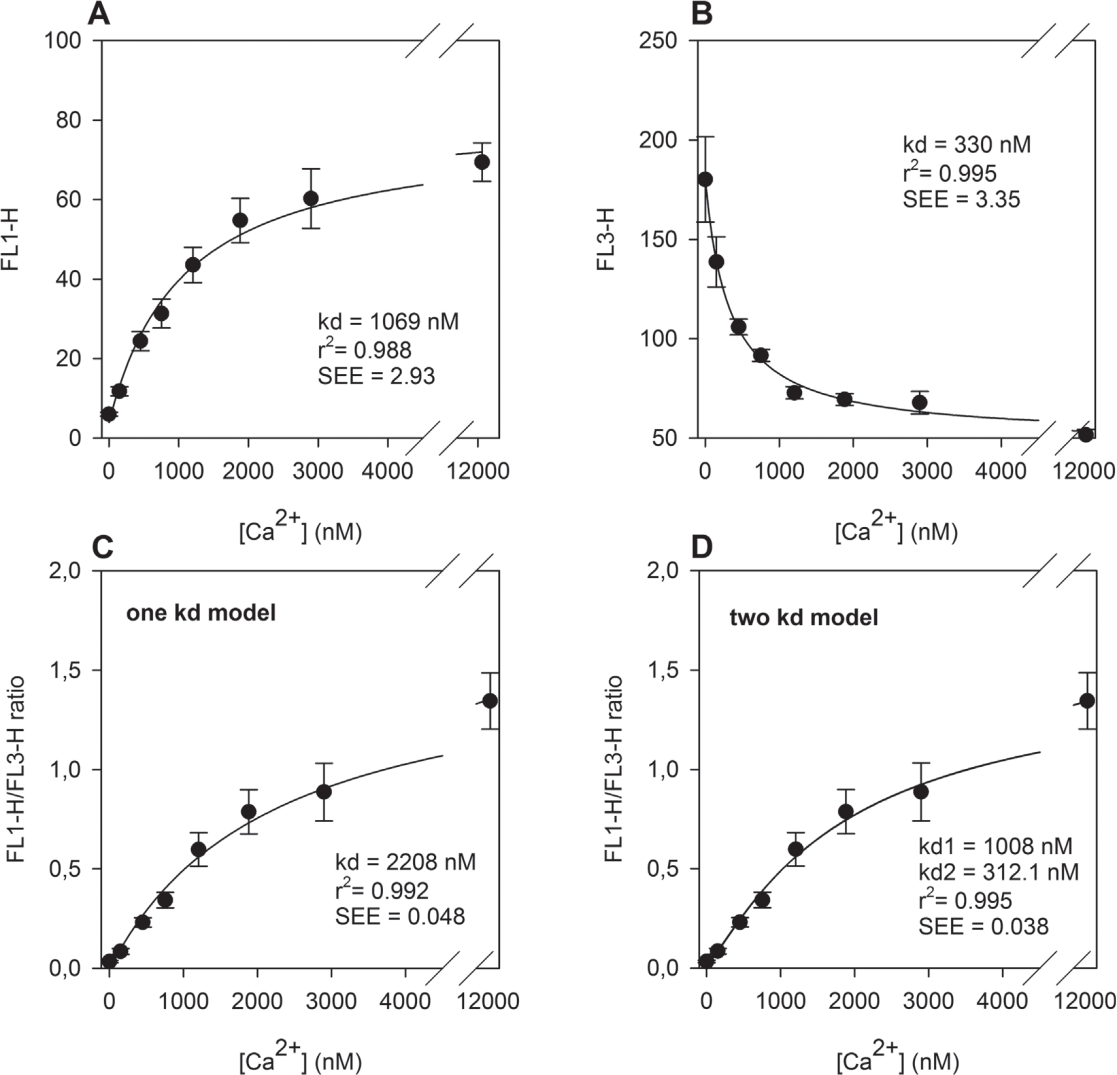
Determination of apparent cellular kd values for Fluo-4 and Fura Red in platelets. **(A)** Fitting of a single kd model (Equation 7A) to mean FL1 data of standard samples (Fluo-4). **(B)** Fitting of a single kd model (Equation 7B) to the mean FL3 data of standard samples (Fura Red). **(C)** Determination of a “mean” cellular kd for both dyes by fitting the “one kd” model (Equation 4) to the mean FL1/FL3 ratios. **(D)** Determination of kd values for Fluo-4 (kd_1_) and Fura Red (kd_2_) by fitting the “double kd model” (Equation 9) for mean FL1/FL3 ratios. The corresponding kd, r^2^ and the standard errors of esteems (SEE) are given as insets. All data are shown as means +/- S.E.M. (n = 6).

First, we determined the fluorescence range of Fluo-4/Fura Red-labelled platelets at different Ca^2+^ concentrations. Therefore, the [Ca^2+^]_i_ concentration was equilibrated with EGTA-containing Ca^2+^-buffers of known free Ca^2+^ concentrations(0 nM—12000 nM) in the presence of ionomycin, CCCP and nigericin. The changes of intraplatelet fluorescence of Fluo-4 and Fura Red at different Ca^2+^ concentrations are depicted in Fig 1A and 1B.

Fitted curves in Fig 1A and 1B were gained by fitting FL1 and FL3 values independent of the obtained data by using Equation 7A and 7B yielding kd_1_ = 1069 nM for Fluo-4 and kd_2_ = 330 nM for Fura Red.

**Fig 2:**
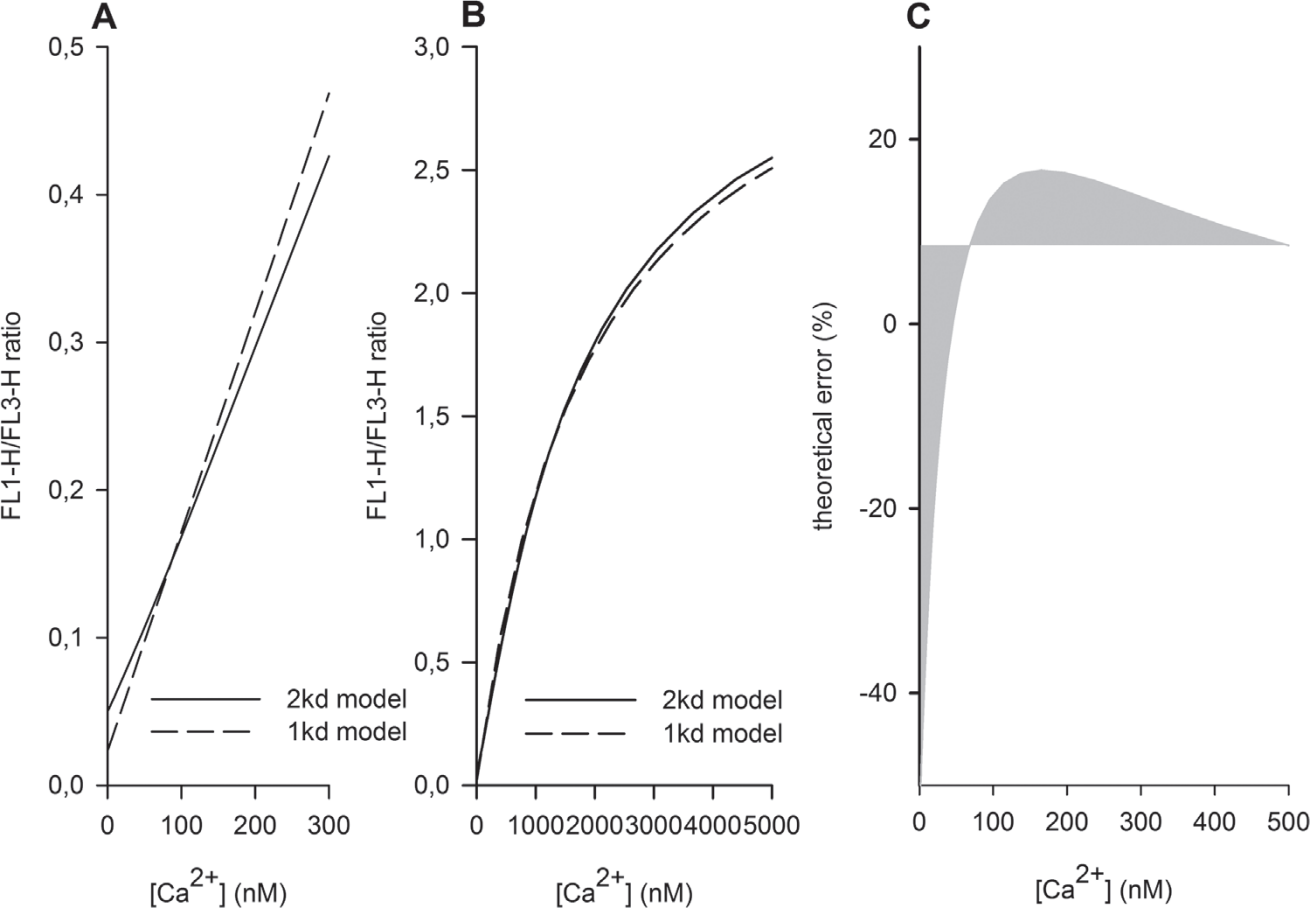
Fluo4/Fura Red ratios calculated by different kd models. Differences between the “one kd” model (Equation 4 [Disp-formula pone.0122527.e007]) and the “double kd model” (Equation 9) for low **(A)** and high **(B)** Ca^2+^ concentrations are shown. **(C)** Theoretical error between the two models is depicted. Both models were fitted by non-linear regression (least squares).

These improved kd_1_ and kd_2_ starting values were used as initial estimates for fitting Equation 10 (“two kd model”) to the ratio/concentration data pairs (depicted in Fig 2D). The final kd_1_ and kd_2_ values were 1008 and 312.1 nM, respectively. The resulting function highly correlated (r^2^ = 0.995) with the obtained data points.

In Fig 1C, FL1/FL3 ratio is correlated to the concentration as a fitted “single kd” function (according to Equation 4, yielding a “combined kd” of 2208 nM), which showed less correlation to the observed data (26% higher standard error estimate) compared to the “double kd” model depicted in Fig 1D.

### Linearity

We further show that ratiometric measurement of [Ca^2+^]_i_ extends the linear range of the measurement. At all [Ca^2+^]_i_ concentrations tested, the ratio FL1/FL3 showed a better linear relationship (r^2^ = 0.9973) to [Ca^2+^]_i_ as solely FL1 (r^2^ = 0.9707) or FL3 (r^2^ = 0.9779) (data not shown). Moreover, the dynamic range (ratio of maximum to minimum signal) of Fluo-4 or Fura Red alone, which was 11.6 and 3.6, respectively, increased to 28.5 when the Fluo-4/Fura Red ratio was employed.

### Theoretical error of a single kd calibration

We then tested whether a “single kd” model with an optimised “mean kd” was able to substitute the “double kd” model (Fig 2). Therefore a “single kd” model (Equation 4 [Disp-formula pone.0122527.e007]) was fitted to the “double kd” model, allowing the variation of the “mean kd” value in this non-linear regression. The single kd model was not able to fully substitute the “double kd” at any “combined kd”. As depicted in Fig 2A, at low Ca^2+^concentrations the “single kd” model yielded in 50% lower FL1/FL3 ratio values compared to the corresponding ratio of the “double kd” model. The theoretical error of the single kd model is shown in Fig 2C. Starting from -50%, around 100 nM, the error crossed the zero line and reached a maximum around 200 nM. At higher concentrations, the relative error, but not the absolute error decreased.

### Ratiometric [Ca^2+^]_i_ measurement is not affected by platelet aggregate formation

**Fig 3:**
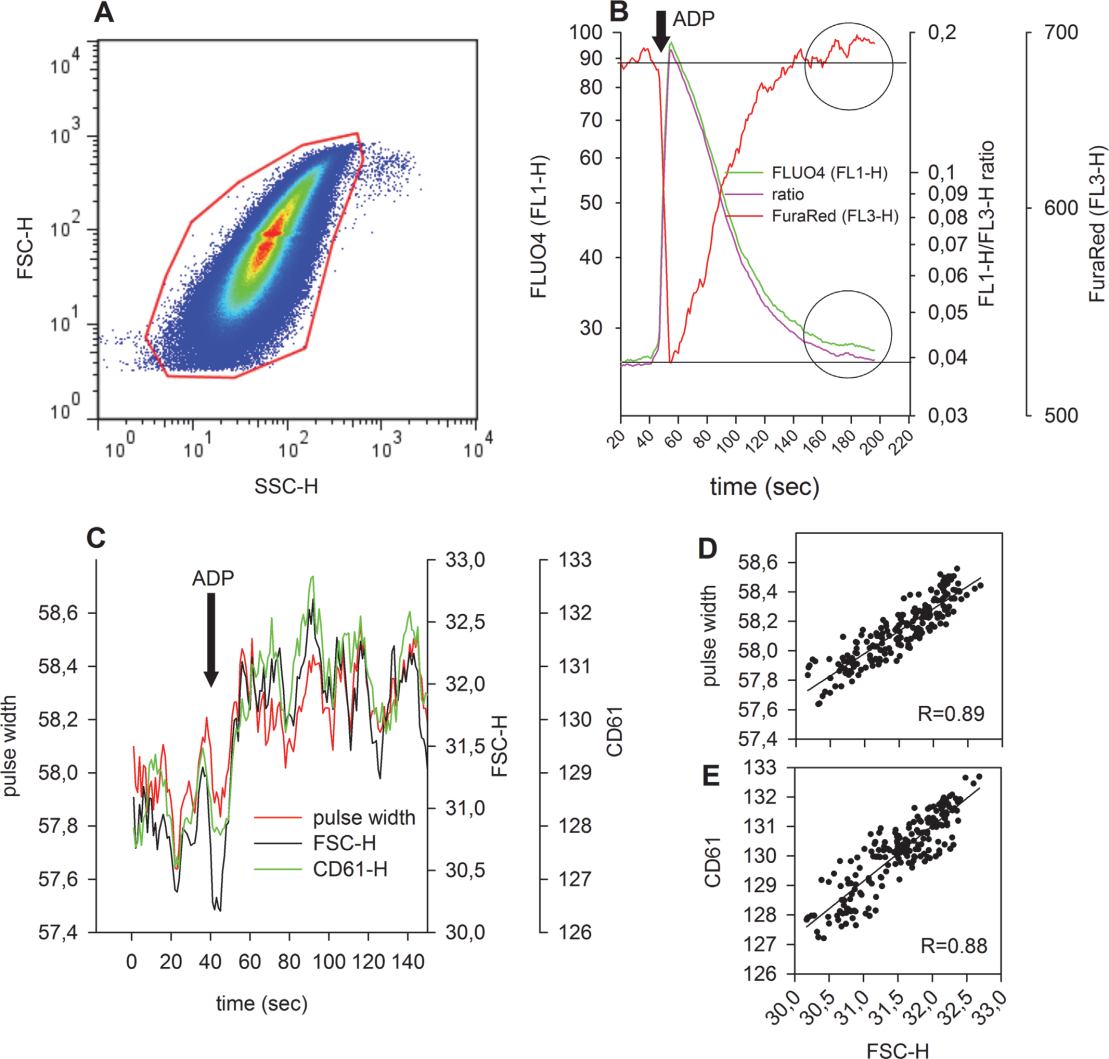
Alterations of detected events in size, pulse width and fluorescence over time: (A) density dot plot of FSC/SSC; red gate = platelet population. **(B)** Time average of FL1 (Fluo-4, green), FL3 (Fura Red, red) and FL1/FL3 ratio (pink) before and after addition of ADP at 45 seconds, as indicated by an arrow. FL1 and FL1/FL3 ratio are depicted in a way as such that basal, as well as maximum responses are super imposable in the graph. Horizontal dotted line represents the basal unstimulated levels of FL1 and FL1/FL3 ratio. Horizontal dashed line shows the basal FL3 level. Two circles indicate the deviation from baseline of FL1 and FL3, respectively, which cannot be seen with the FL1/FL3 ratio, about 120 seconds after ADP stimulation. **(C)** Time averaging of FSC, pulse width and CD61 (FL4) events (medians). Addition of ADP is indicated by an arrow. **(D)** Correlation between pulse width and FSC during ADP stimulation in platelets. **(E)** Correlation between CD61 (FL4) and FSC during ADP stimulation in platelets. In (D) and (E) the solid line illustrates the correlation between the parameters as a linear regression line. The corresponding correlation coefficients are given as insets.

We then determined if this ratiometric measurement could overcome artefacts due to increased fluorescence signals caused by platelet aggregation. Whole blood labelled with anti-CD61, Fluo-4 and Fura Red was gated for platelets (Fig 3A, red gate). After 45 seconds of measurement, 100 μM ADP was added and the changes in forward scatter (FSC), pulse width and platelet-specific fluorescence (CD61) analysed over time (Fig 3B and 3C). Stimulation with ADP caused a permanent increase in the FSC (approximately 8%) and was accompanied by a similar increase in pulse width and CD61 fluorescence (FL4), indicating that the formation of platelet aggregates caused an increase in fluorescence compared to single platelets.

Time course of fluorescence signals in FL1 and FL3 as well as FL1/FL3 ratios are depicted in Fig 3B and show that ADP stimulation results in a permanent increase in FL1 fluorescence, which is not apparent when data are calculated as ratio. Moreover, fluorescence signals in FL3 even exceeded the basal level, which also reflects a consequence of platelet aggregate formation.

To confirm that increased fluorescence (caused by changes of event size) affects FL1 and FL3 to a similar extent, we compared platelets in the presence of different Ca^2+^concentrations for their fluorescence in FL1 and FL3.

**Fig 4:**
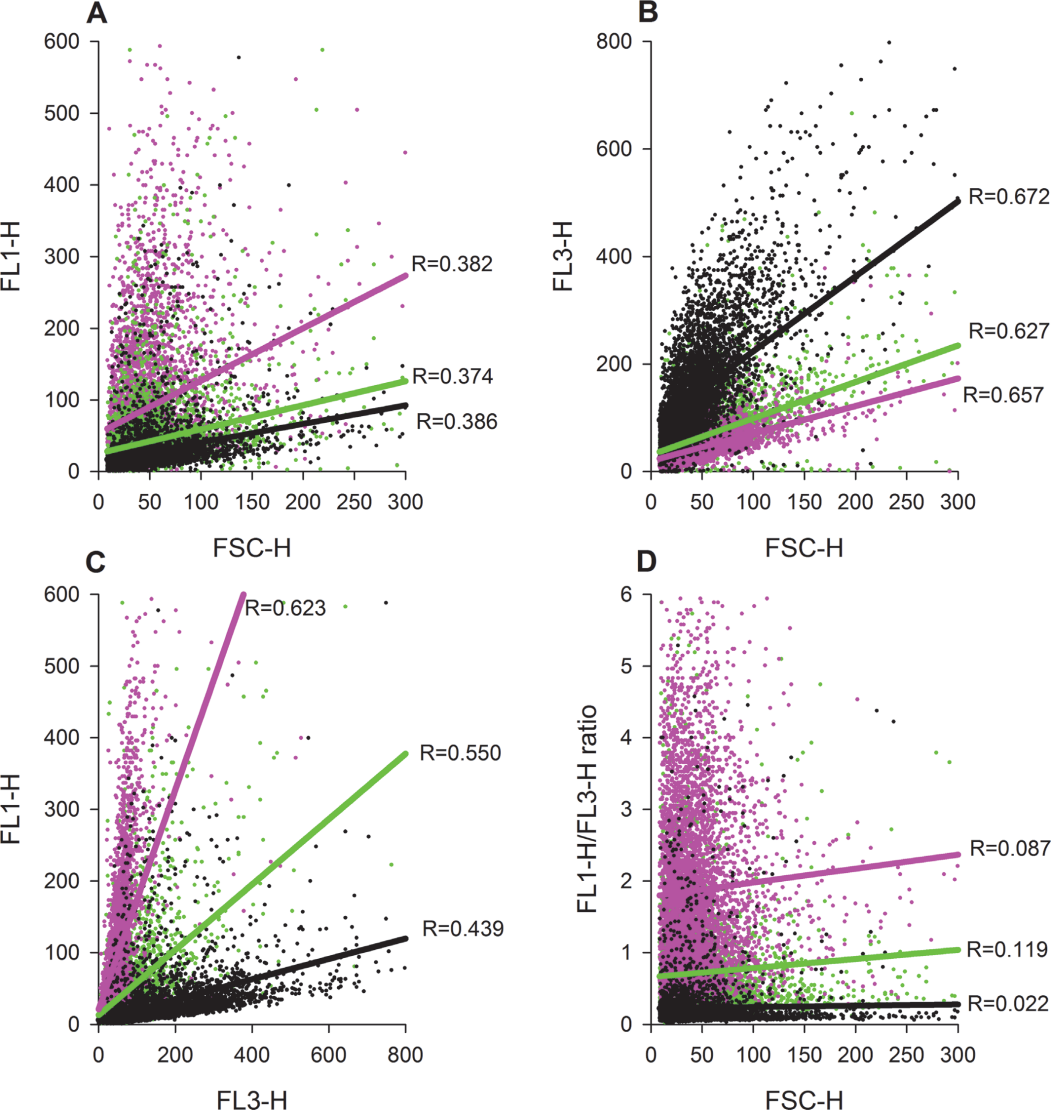
Correlations between Fluo-4 and Fura Red fluorescence with event size at three different Ca^2+^ concentrations (0, 413 or 1012 nM free Ca^2+^): (A) Correlation between Fluo-4 fluorescence (FL1) and event size (FSC). **(B)** Correlation between Fura Red fluorescence (FL3) and event size (FSC). **(C)** Correlation between Fluo-4 fluorescence (FL1) and Fura Red fluorescence (FL3).**(D)** Correlation between Fluo-4/Fura Red (FL1/FL3) ratio and event size (FSC). Black = Ca^2+^ free buffer; green = 413 nM free Ca^2+^; magenta = 1012 nM free Ca^2+^. In all subfigures the regression coefficient R are given as insets for each Ca^2+^ concentration used.

Fig 4A and 4B show the correlation between event size (FSH) and fluorescence FL1 (r = 0.374–0.386) and FL3 (r = 0.627–0.672) at three different Ca^2+^ concentrations. FL1 correlated with FL3 (r = 0.439–0.623) and depended on [Ca^2+^]_i_ (Fig 4C). In contrast to FL1 and FL3, the FL1/FL3 ratio did not correlate with FSC (Fig 4D), indicating that the ratio is independent of the event size.

From these data we conclude that formation of platelet aggregates affects single channel fluorescence even under constant [Ca^2+^]_i_ conditions, while the fluorescence ratio is independent of the event size (i.e., cell size and/or aggregate formation). In cases where formation of platelet-platelet aggregates occurs during time-dependent flow cytometry, the acquisition of fluorescence ratios would therefore prevent misinterpretation of measured parameters that derive from changes in event size.

### Impact of ADP on Ca^2+^ reuptake rate after platelet stimulation

To determine whether ADP stimulation of platelets influences the reuptake kinetics of Ca^2+^ into intracellular stores represents a demanding task as critical technical requirements must be met for such experiments: (i) cell aggregate formation must not influence the readout for Ca^2+^, (ii) a high dynamic linear measurement range must be provided so that the reuptake rate at different actual intracellular Ca^2+^levels does not cause any bias.

**Fig 5:**
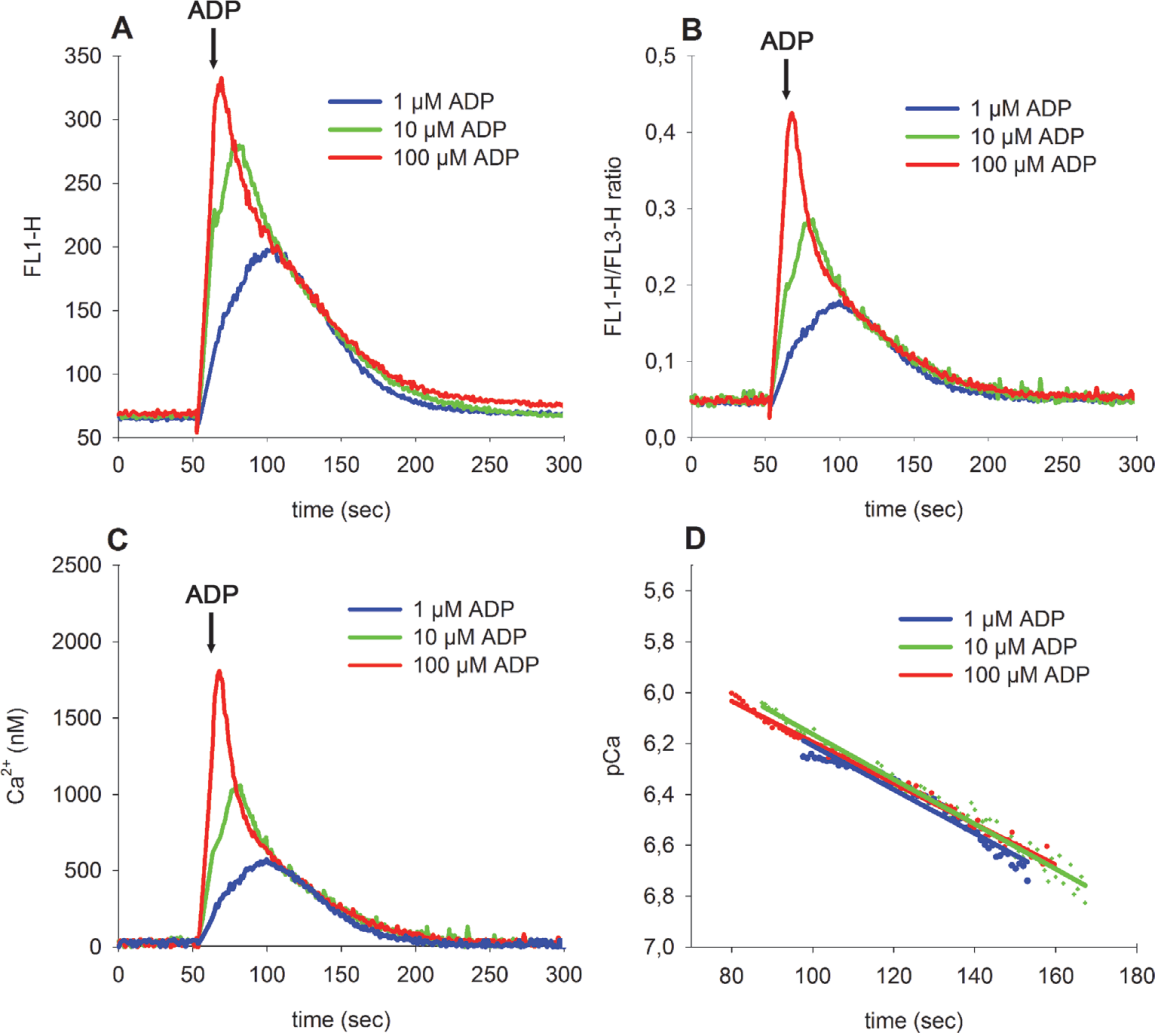
The effect of platelet activation by different concentrations of ADP (1–100 μM) on intraplatelet [Ca^2+^]_i_ dynamics over time. **(A)** Time course of time-averaged FL1-fluorescence. **(B)** Time course of time-averaged FL1/FL3 ratios. **(C)** Time course of [Ca^2+^] transformed from FL1/FL3 ratios using Equation 10A and 10B (see Methods Section). **(D)** Log_10_-transformed ratios in the descending part of traces from subfigure C are shown as dots. Linear regression lines in the same colour were fitted to these data points. ADP was added 50 seconds after initial recording as indicated by an arrow.

To demonstrate that the described method fulfils these requirements, we measured [Ca^2+^]_i_ before, during and after stimulation of platelets with 1, 10 and 100 μM ADP (Fig 5). While the FL1-signal (Fig 5A) did not completely return to baseline levels after stimulation with 100 μM ADP, the ratio of FL1/FL3 reached basal levels 200 seconds after ADP stimulation (Fig 5B), indicating that FL1 is prone to artefacts due to platelet aggregate formation. After transforming ratios to Ca^2+^-values as shown in Fig 5C, the reuptake traces were transformed by a logarithmic function to obtain first order reuptake rates represented by the slopes (Fig 5D). The resulting reuptake rates were equal and independent of ADP concentration, indicating that the degree of platelet activation does not influence the reuptake process, which is assumed to be a first order elimination process. Thus, application of the “double kd” transformation results in accurate and undistorted Ca^2+^-values over a wide dynamic range.

### Protocol for a rapid implementation of the described method

**Fig 6:**
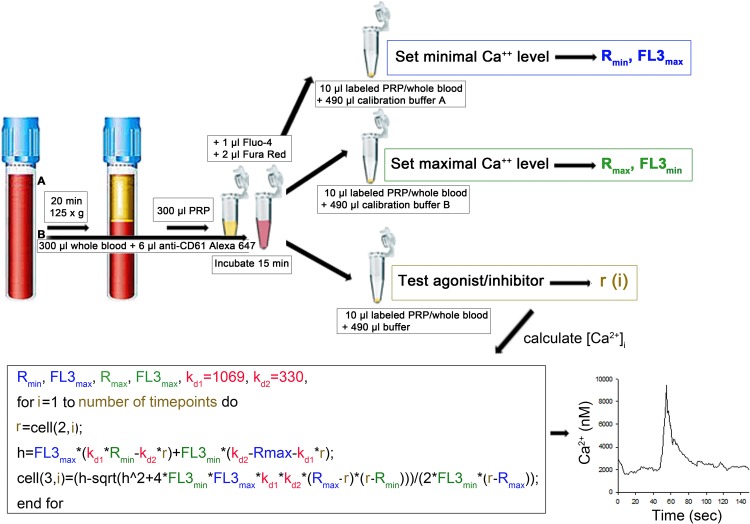
Overview of the test procedure. **(A)** PRP or **(B)** citrated whole blood can be used for the assay. Whole blood has to be incubated with anti-CD61 antibody to allow detection of platelets. 300μl of the respective cell suspension is incubated with 1μl Fluo-4 (1mg/ml) and 2 μl Fura Red (1mg/ml) for 15 min at 37°C and 5% CO_2_ at dark. 10 μl of cell suspension are then transferred into FACS tubes containing 490μl calibration buffer A (10 mM CaEGTA, 3 μg/ml ionomycin, 2 μg/ml nigericin and 10 μM CCCP) or calibration buffer B (10 mM K_2_EGTA, 3 μg/ml ionomycin, 2 μg/ml nigericin and 10 μM CCCP), respectively. 10 μl PRP or whole blood diluted in 490 μl HEPES-Tyrode buffer can then be used for each sample analysis. Basal levels of [Ca^2+^]_i_ should be recorded for approximately 50 seconds, followed by the addition of the agonist and further recording for changes in FL1 and FL3 over at least a further 100 seconds. This will yield in FL1/FL3 ratios over time (r_i_), which can be calculated e.g. by FlowJo and allows calculation of absolute [Ca^2+^]_i_ levels by the depicted algorism in e.g. Sigma Plot given that time data are exported in the 1st column and FL1/FL3 ratios into the 2nd column. Blue values (R_min_ and FL3_max_) are obtained by calibration in Ca^2+^ free buffer, green values (R_max_ and FL3_min_) are obtained by measuring maximal calcium concentrations, golden values (r, i) are obtained from the actual sample measured and red values (kd1 and kd2) represent values that have been determined within this manuscript.

Fig 6 describes the test procedure to calibrate and measure [Ca^2+^]_i_. Experiments can either be performed in diluted PRP (A) or whole blood (B) and only 2 calibration measurements in control buffers are required to set the minimal and the maximal calcium signal to calculate [Ca^2+^]_i_ in any sample by the algorithm given in Fig 6.

## Discussion

In this study we describe a novel method to quantify intraplatelet [Ca^2+^]_i_ dynamics by ratiometric flow cytometry using Fluo-4 and Fura Red. We provide equations to easily calculate [Ca^2+^]_i_ levels in a 2-kd model as well as a ready-to-use protocol to quantify intraplatelet [Ca^2+^]_i_ dynamics in whole blood and PRP by flow cytometry.

Flow cytometry is based on acquisition of single “cellular” events according to their optical properties such as light scatter and/or fluorescence intensity. However, platelet activation coincides with the formation of platelet-platelet aggregates, and these aggregates are identified as single events by flow cytometry. Consequently, aggregate formation results in artificially elevated signals as total fluorescence arising from cell aggregates is increased compared to single platelets. We are able to demonstrate that aggregate formation affects fluorescence in all detection channels to a similar extent. Therefore, our method of combining two dyes that either increase (Fluo-4) or decrease (Fura Red) in fluorescence upon binding of[Ca^2+^]_i_ can prevent such artefacts and the obtained results appear to be independent of cell/aggregate size and are therefore unaffected by platelet aggregate formation. Moreover we show that physiological concentrations of divalent ions, like magnesium (Mg^2^), zinc (Zn^2+^) and manganese (Mn^2+^), which also bind to Fluo-4 and Fura Red, do not significantly interfere with [Ca^2+^]_i_ measurement (see S1 File).

Thus, our method provides essential advantages compared to single dye models, which display increased fluorescence due to formation of platelet-platelet aggregates. Fluo-4/Fura Red ratio further showed more accurate results in terms of linearity and dynamic range compared to single fluorescence measurements.

Of note, it has been previously reported that both the efflux and the photobleaching rates of Fluo-4 and Fura Red are almost equal [12], making these two dyes a perfect combination for ratiometric measurement.

Previously published ratiometric methods to quantify [Ca^2+^]_i_ dynamics were only described for Indo-1, a dye that does not allow experiments with compounds that display a disturbing fluorescence emission in response to UV-light (e.g., oxidised lipoproteins, oxidised phospholipids, polyaromatic compounds). In addition, Indo-1 shows a spectral overlap with NADH/NADPH autofluorescence and is therefore affected by variations in energy metabolism—furthermore, it bleaches more rapidly compared to Fluo-4 and Fura Red.

Fluo-4 is a Fluo-3 analog with two chlorine substituents replaced by fluorines. Due to this structural modification fluorescence excitation at 488 nm is increased in Fluo-4 compared to Fluo-3 and consequently leads to stronger fluorescence signals, which makes Fluo-4 an attractive tool for measuring [Ca^2+^]_i_ dynamics by flow cytometry, confocal microscopy and microplate screening applications[13]. Moreover Fluo-4 is less prone to photobleaching compared to Fluo-3 (with a t_1/2_ of 339 s^-1^ versus 143 s^-1^respectively). There are no significant differences between Fluo-3 and Fluo-4 in intracellular accumulation [12], but Fluo-3 has a higher dynamic range compared to Fluo-4 [14].

Calcium-sensitive dyes are usually applied as membrane-permeable acetoxy-methyl ester derivatives (AM-esters). Cellular loading and accumulation of these dyes results from cleavage of these esters by unspecific intracellular esterases that convert the lipophilic (pro)dyes into polar membrane-impermeable forms that thereby also acquire the ability to bind Ca^2+^. The fact that the Fluo-4/Fura Red AM-esters show virtually no fluorescence represents another important advantage to Indo-1, which possibly underestimates intraplatelet [Ca^2+^]_i_ due to the presence of residual (non Ca^2+^ responsive) AM-esters, which associate with the membrane and/or were not washed away [7].

AM esters neutralize the charges of fluorescent dyes and indicators, allowing passive diffusion into live cells. The indicators are then converted by intracellular esterases into a cell-impermeant dye that is retained inside the cell.AM esters also enter organelles. While there are no significant differences between Fluo-3 and Fluo-4 in intracellular accumulation, compartimentalisation is higher in Fluo-4 compared to Fluo-3. Moreover, Fluo-4 shows a higher accumulation in the nucleus compared to Fluo-3 [12], but since platelets have no nucleus this is not a problem in this experimental setting. Compartimentalisation of these dyes can lead to a slight slowing of recovery rates and increased baseline at high stimulation rates[14]. Due to their similar nature both Fluo-3 or Fluo-4 can be used for quantitiative, ratiometric intraplatelet [Ca^2+^]_i_ dynamics.

We are able to show that application of Fluo-4/Fura Red ratio is suitable for measurement of basal intraplatelet [Ca^2+^]_i_ as well as for intraplatelet [Ca^2+^]_i_ dynamics.

Basal and maximal Fluo-4 and Fura Red fluorescence levels need to be determined for each individual experiment as the intracellular abundance of compounds that interfere with concentrations of [Ca^2+^]_i_ depends on experimental conditions and furthermore might differ between individual donors. Therefore, to quantify [Ca^2+^]_i_ dynamics calibration with only 2 buffers, a calcium free and a high calcium (10 mM CaEGTA) buffer, is necessary. As active calcium transport processes might impose gradients or affect equilibration, CCCP needs to be added as a metabolic inhibitor of mitochondria in a glucose-free buffer [15]. Further, impaired mitochondrial energy metabolism might result in intracellular acidification and would thereby influence the binding constant of EGTA for calcium and thus the free calcium concentration inside the cell. To avoid this bias, we further added nigericin, a hydrogen-ion ionophore [16] to our sample.

For ratiometric Ca^2+^ indicators like Fura-2 or Indo-1, appropriate equations to calibrate the fluorescence and calculate quantitative Ca^2+^ levels using a “single kd” model have been provided [5]. However, we can show that “single kd” equations are not suitable for ratio levels obtained by two independent fluorescence dyes. Therefore, we implemented the first “double kd” model, which shows a higher accuracy and a higher dynamic range.

In our cellular setting, the apparent kds of fluorescent dyes for calcium were higher compared to binding constants measured in homogenous cell free solutions, presumably due to calcium binding molecules like calmodulin that are present in the cytosol and due to the decreased pH inside of platelets, resulting in higher apparent kds due to the elevated number of interfering H^+^ ions.

Taken together we describe a novel, rapid method to quantify intraplatelet [Ca^2+^]_i_ dynamics in whole blood or PRP, which yields results that are unaffected by platelet aggregate formation using flow cytometry. This method can potentially be extended to other applications like live cell microscopy under flow conditions. Further research is necessary to determine if the described kds are also suitable for flow-based immunofluorescence microscopy.

## Supporting Information

S1 File (DOCX)
