# Supplementary material for: A Novel, Rapid Method to Quantify Intraplatelet Calcium Dynamics by Ratiometric Flow Cytometry
Source: PLoS One. 2015 Apr 7;10(4):e0122527. doi: 10.1371/journal.pone.0122527 (PMC4388375; doi:10.1371/journal.pone.0122527)
Supplement: S1 File — (DOCX) [file pone.0122527.s001.docx]

**S1 File**

## Impact of metal ions on [Ca^2+^]_i_ measurement

Fluo-4 and Fura Red are not specific for Ca^2+^ but also sensitive to divalent heavy metal cations like manganese (Mn^2+^) and zinc (Zn^2+^) which bind Fluo-4 and Fura Red with a higher affinity than Ca^2+^. Compared to physiological plasma concentrations of Ca^2+^, physiological Zn^2+^ and Mn^2+^ concentrations are rather low. Zn^2+^ is found at plasma concentrations of 10-17  µM and Mn^2+^ at plasma concentrations of 5 –20 nM. To determine if Zn^2+^ and Mn^2+^ interfere with [Ca^2+^]_i_ measurement we added low and high physiologically relevant concentrations of Zn^2+^ and Mn^2+^ to the calibration buffers and determined the interference of Zn^2+^ and Mn^2+^ with Fluo-4 and Fura Red signals (Figure 1).

Another ion that potentially alters Fluo-4 and Fura Red signals is magnesium (Mg^2+^). Compared to Zn^2+^ and Mn^2+^, Mg^2+^ is found at much higher plasma concentrations, which range between 0.7 and 1.2 mM. Mg^2+^ is known to inhibit platelet functions [1-3] but its influence on calcium signals by Fluo-4 and Fura Red has not been evaluated. We determined the kd of Fluo-4 and Fura Red in the absence of Mg^2+^ and evaluated if hypo- (0.5mM) and hyper-magnesemic (1.2mM) conditions influence fluorescence intensities of Fluo-4 and Fura Red in calibration buffer A and B.

As depicted in Figure 1, none of the tested ions led to an increase in basal fluorescence levels (buffer A), nor did the presence of these divalent ions provoke a significant change in the increased Fluo-4/Fura Red ratio in the presence of calcium.

From these data we conclude that physiological concentrations of Mg^2+^, Mn^2+^ and Zn^2+^ do not significantly alter the fluorescence signals of the calcium sensitive dyes Fluo-4 and Fura Red.

Mg^2+^ itself alters [Ca^2+^]_i_ signals due to its direct platelet inhibiting effects, but we can rule out that Mg^2+^ interferes with [Ca^2+^]_i_ detection in our experimental setting. Mn^2+^ and Zn^2+^ have direct platelet activating effects independent of modulation of Fluo-4 and Fura Red fluorescence signals [4]. Therefore they might directly alter [Ca^2+^]_i_ levels but do not interfere with the measurement itself.


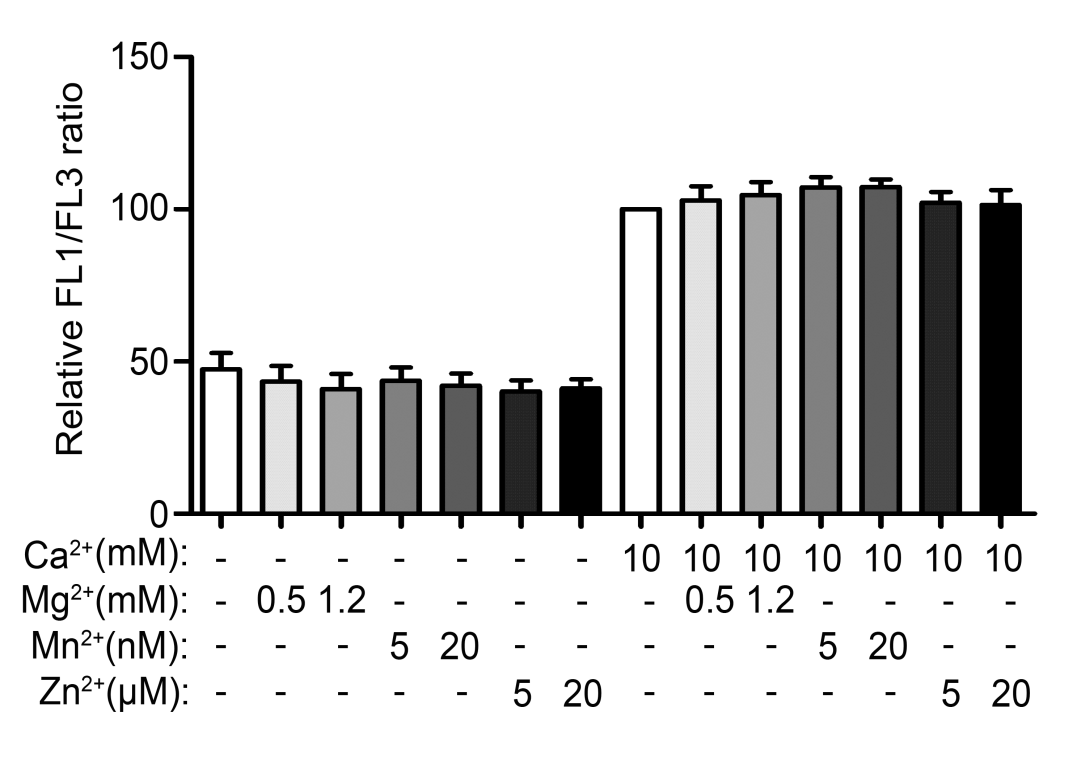


**Figure 1: Effect of metal ions on Fluo-4 and Fura Red signals.** To determine if Mg^2+^, Zn^2+^, and Mn^2+^ interfere with Fluo-4 and Fura Red signals we determined FL1 and FL3 in calibration buffers in the presence and absence of sub- and supra-physiological concentrations of these ions (Mg^2+^: 0.5-1.2mM; Zn^2+^: 5-20µM; Mn^2+^: 5-20nM). Mean and SEM of 5 independent experiments.

**References**

1. Ravn HB, Kristensen SD, Vissinger H, Husted SE (1996) Magnesium inhibits human platelets. Blood Coagul Fibrinolysis 7: 241-244.

2. Ravn HB, Vissinger H, Kristensen SD, Husted SE (1996) Magnesium inhibits platelet activity--an in vitro study. Thromb Haemost 76: 88-93.

3. Ravn HB, Vissinger H, Kristensen SD, Wennmalm A, Thygesen K, et al. (1996) Magnesium inhibits platelet activity--an infusion study in healthy volunteers. Thromb Haemost 75: 939-944.

4. Marx G, Krugliak J, Shaklai M (1991) Nutritional zinc increases platelet reactivity. Am J Hematol 38: 161-165.
